# Persistent warm Mediterranean surface waters during the Roman period

**DOI:** 10.1038/s41598-020-67281-2

**Published:** 2020-06-26

**Authors:** G. Margaritelli, I. Cacho, A. Català, M. Barra, L. G. Bellucci, C. Lubritto, R. Rettori, F. Lirer

**Affiliations:** 1 Istituto di Ricerca per la Protezione idrogeologica (IRPI), CNR, via della Madonna Alta 126, 06128 Perugia, Italy; 2 GRC Geociències Marines, Dept. de Dinàmica de la Terra i de l’Oceà, Facultat de Ciències de la Terra, Universitat de Barcelona, Barcelona, Spain; 3 Istituto di Scienze Marine (ISMAR), CNR, Calata Porta di Massa, Interno Porto di Napoli, 80133 Napoli, Italy; 4 Istituto di Scienze Marine (ISMAR), CNR, Via Gobetti 101, 40129 Bologna, Italy; 5 Dipartimento di Scienze e Tecnologie Ambientali Biologiche e Farmaceutiche (DiSTABiF), Università della Campania “Luigi Vanvitelli”, Via Vivaldi 47, Caserta, Italy; 6 Dipartimento di Fisica e Geologia, Università degli Studi di Perugia, Via Alessandro Pascoli, 06123 Perugia, Italy

**Keywords:** Palaeoceanography, Palaeoclimate, Climate sciences, Environmental sciences, Ocean sciences

## Abstract

Reconstruction of last millennia Sea Surface Temperature (SST) evolution is challenging due to the difficulty retrieving good resolution marine records and to the several uncertainties in the available proxy tools. In this regard, the Roman Period (1 CE to 500 CE) was particularly relevant in the socio-cultural development of the Mediterranean region while its climatic characteristics remain uncertain. Here we present a new SST reconstruction from the Sicily Channel based in Mg/Ca ratios measured on the planktonic foraminifer *Globigerinoides ruber*. This new record is framed in the context of other previously published Mediterranean SST records from the Alboran Sea, Minorca Basin and Aegean Sea and also compared to a north Hemisphere temperature reconstruction. The most solid image that emerges of this trans-Mediterranean comparison is the persistent regional occurrence of a distinct warm phase during the Roman Period. This record comparison consistently shows the Roman as the warmest period of the last 2 kyr, about 2 °C warmer than average values for the late centuries for the Sicily and Western Mediterranean regions. After the Roman Period a general cooling trend developed in the region with several minor oscillations. We hypothesis the potential link between this Roman Climatic Optimum and the expansion and subsequent decline of the Roman Empire.

## Introduction

The semi-enclosed configuration of the Mediterranean Sea makes this an extremely vulnerable region to modern and also to past climate changes^1^.The strategic transitional zone that occupies the Mediterranean, between North Africa and European climates, from the arid zone of the subtropical high to the humid northwesterly air flows, provides to the Mediterranean a particular interest to unravel climate tele-connections during times of climate variability^2–4^. Several studies carried out in different marine sites, have focused the attention to the short-term climate variability over the last millennia but so far, any general reconstruction of the regional temperature evolution has not been attained^5–12^. Compared to other regions of the world, the Mediterranean is characterised by a wealth of archaeological studies and historic documents, as well as a paleoclimatic data that makes it a perfect case study to investigate the potential influence of climate on civilisations^13^. In fact, this time period is particularly challenging since coincided with important cultural changes (human civilizations) that developed around the Mediterranean area^14^. The study of the fossil archives remains the only valid tool to reconstruct past environmental and climatic changes during those times^15^. However, its application in the marine realm is compromised by the difficulty to obtain marine records with resolution enough, and clear proxy signals that can be reproduced among different records. Climate variability of this period is often close or within the proxy errors and uncertainties in its interpretation in terms of seasonality and/or local oceanographic processes, make difficult always its reading in terms of regional climate evolution^11,12,16–18^. Nevertheless, this is a critical information to identify past interactions between climate changes and evolution of human societies and their adaptive strategies^19–25^. In addition, the last report of the Intergovernmental Panel on Climate Change (IPCC 2018) underlines the requirement to assess climate feedbacks during past episodes of moderately warmer (1.5–2 °C) conditions^26,27^.

In this framework, we present a new generated SST record reconstruction from the central part of the Mediterranean Sea based on Mg/Ca ratios measured in the planktonic foraminifera *Globigerinoides ruber* covering the last 5 kyr BP. This new record is compared with other previously published SST records from the Alboran Sea, Menorca Basin and Aegean Sea based on different geochemical proxies and with a reconstruction of the NAO index^28^. We argue that this trans-Mediterranean compilation provides the basis to discuss main SST features during the last two millennia focussing the attention into the Roman period since it underscores as the warmest period with interesting implications on civilization development in the Mediterranean region.

## Study area

The Mediterranean Sea is an anti-estuarine semi-enclosed sea that can be subdivided into two sub-basins, the western and eastern Mediterranean separated by the Sicily Strait sill^29^. Low salinity surface waters, called Modified Atlantic Water (MAW), enter in the Mediterranean Sea from the Strait of Gibraltar and occupy the first 0–100 m of the water column which overlies the outflowing saltier Levantine Intermediate Water (LIW) formed in the Levantine basin^29^. The MAW entrained by an intense jet coupled to a wave-like front^30,31^, cross the Alboran Sea and exits in its eastern end along the African coast^32^.

**Figure 1:**
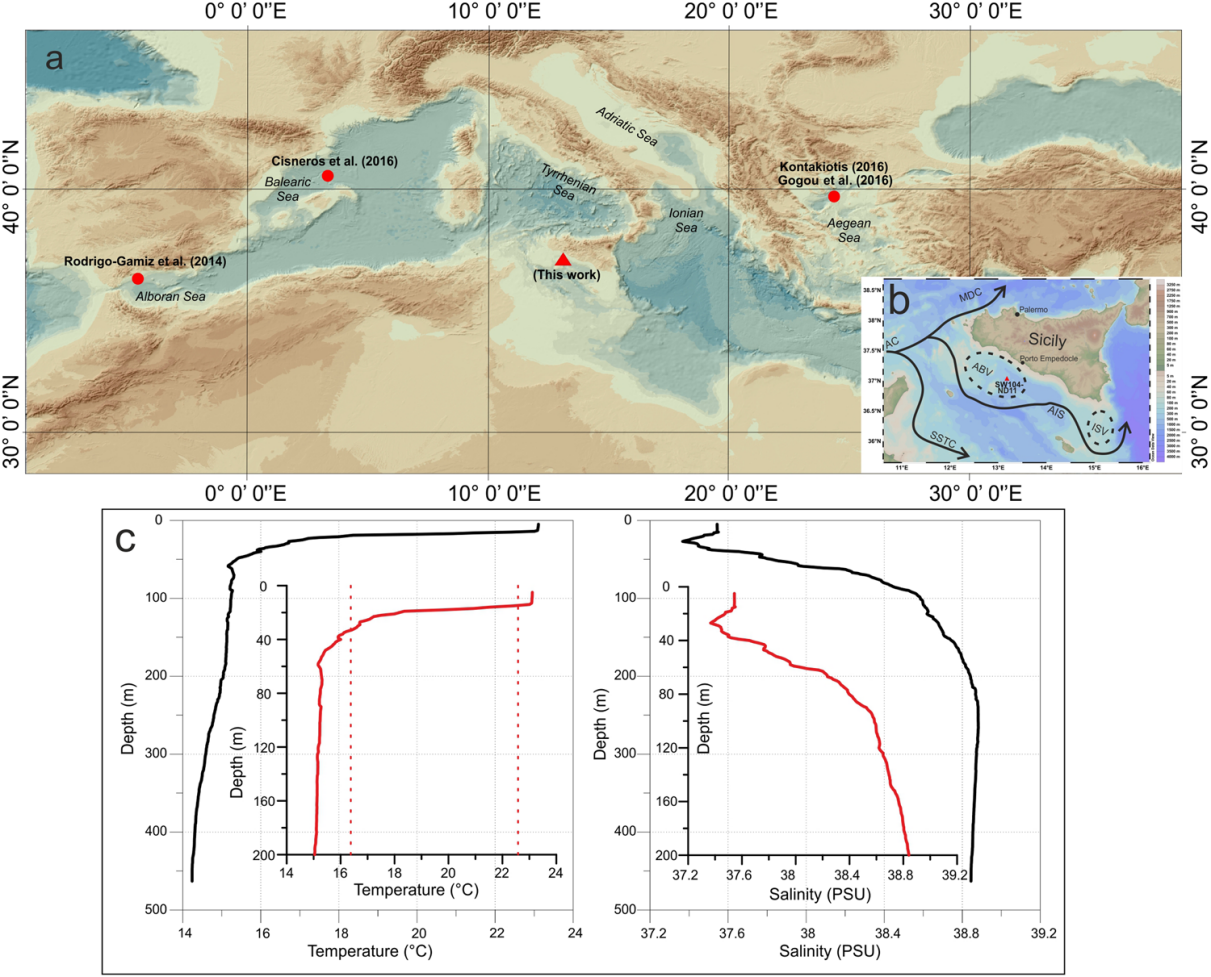
(**a**) Bathymetric map of the central-western Mediterranean Sea. The background map is the GEBCO global terrain model for ocean and land (15 arc-second intervals, World Geodetic System, WGS-84 datum; https://www.gebco.net/). Red triangle: location of SW104-ND11 core; red circles: marine records used for the comparison. (**b**) Bathymetric map of the Sicily Channel has been generated using Ocean Data View according to the reference reported in http://odv.awi.de showing surface oceanographic circulation and core location. Black lines follow the path of surface water circulation. Major currents are illustrated. AC, Atlantic Current; SSTC, Sicily Strait Tunisian Current; AIS, Atlantic Ionian Stream. ABV, Adventure Bank Vortex; ISV, Ionian Shelfbreak Vortex; MDC, Middle Tyrrhenian Current^29^. Red triangle: location of SW104-ND11 core. **(c) **Profiles of selected physical seawater parameters (salinity and temperature) from study core SW104-ND11 in July 2014. The vertical dotted lines on the temperature plot represent the range of temperature values represented by the Mg/Ca_*G.ruber*_ record for the SW104-ND11 core.

MAW flows along the Algerian coast as the coastal Algerian Current and separates into two branches at the entrance of the Sicily Strait^33^ (Fig. 1). The Strait of Sicily represents a physical barrier (about 500 m deep) of the eastern Mediterranean and implies considerable control over the biogeochemicals processes occurring within the eastern basin^34^. Most of the MAW passes through the Sicilian Channel^35^ dividing into two streams while the rest flows into the Tyrrhenian Sea^36,37^. The northern branch, called the Atlantic Ionian Stream (AIS), forms the MAW transport into the eastern Mediterranean off the southern coast of Sicily and baths the studied location^38^.

In detail, measurements of the main physical seawater parameters on the studied core location (SW104-ND11) in July 2014 (Fig. 1) show that surface temperatures (0–15 m) range from 23.0 °C to 22.0 °C. The base of the thermocline (17.5 °C) is located at a depth of 22 m and the salinity values are between 37.36 and 38.88 psu.

## Results and discussion

**Figure 2:**
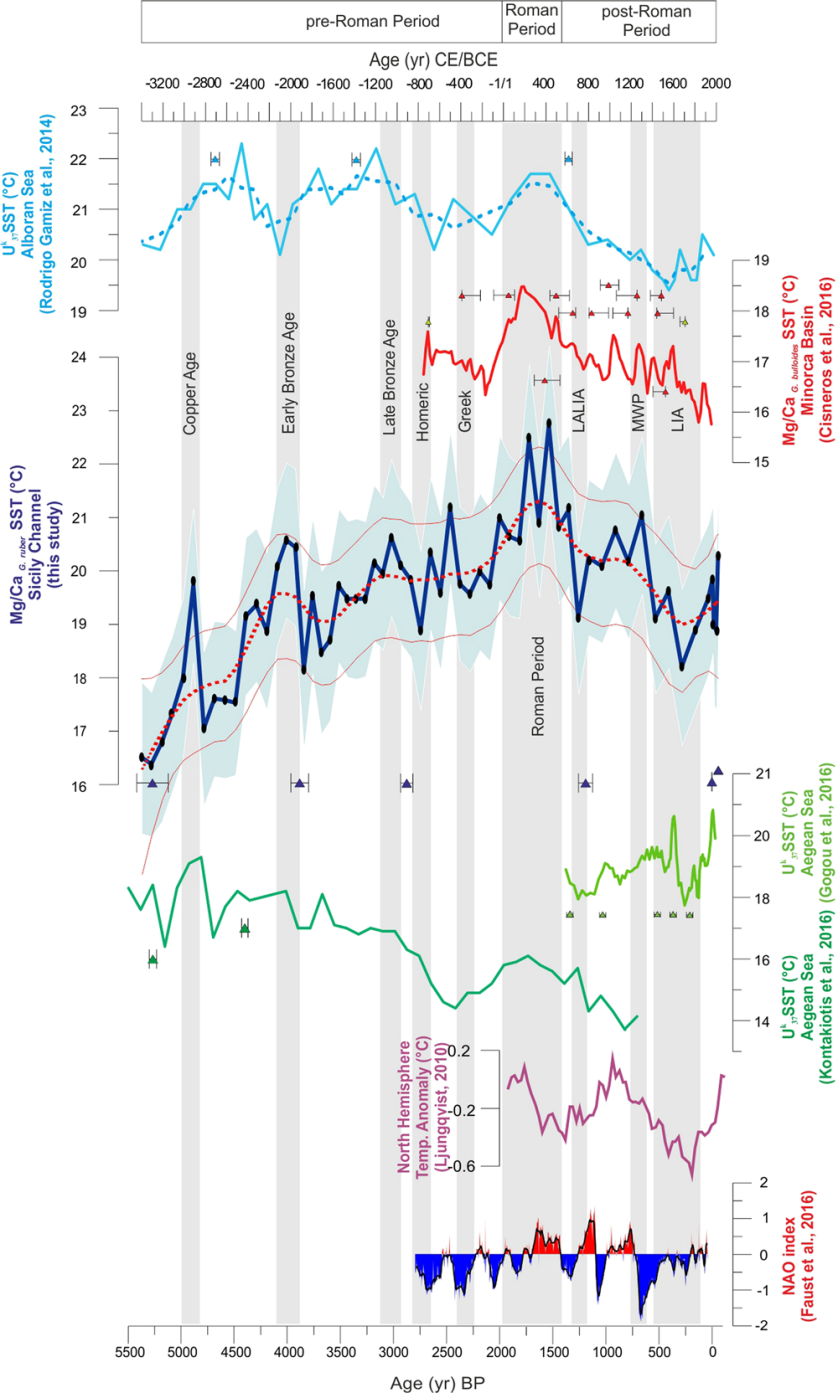
Comparison in time domain of the SSTs records from Sicily Channel (Mg/Ca _*G.ruber*_ core SW104-ND11, this work), Alboran Sea^39^, Minorca Basin^16^, Aegean^40,41^ with the north Hemisphere temperature reconstruction^42^ and NAO index^28^. The thick dashed line superimposed to the SST raw data in Alboran Sea^39^ is 3 points running average; the red dashed line of the Sicily Channel record (this study) represents the 95% CI smoothed curve (Monte Carlo simulation) and the thin red lines are the 2.5% and the 97.5% CI of 10000 LOESS fitted realizations of the data (see Material and Methods paragraph). The black dots over the Sicily Channel record (blue curve) represent analysed data and the light blue shadow is the propagation error. The grey bands show the main climate events documented in the Mediterranean basin and discussed in the text (e.g. Roman Period^62^). Close to each SST record are the dating points with the associated error bars.

The SST estimates from the Mg/Ca_*G.ruber*_ ratio range from 16.4 °C ± 1.5 °C to 22.7 °C ± 1.5 °C with a mean value of 19.5 °C ± 1.5 °C (Fig. 2); to document the main trends of variability [95% Confidence Interval (CI)] in SST reconstruction we adopted a Monte Carlo approach that use a non-parametric regression (LOESS function, see Material and Methods paragraph). The Mg/Ca_*G.ruber*_ SST reconstruction from core SW104-ND11 shows a progressive warming trend of 6.3 °C ± 2.0 °C from 3300 BCE (base of the sequence) to 330 CE, middle Roman Period when SST maxima are attained (Fig. 2). This long-term warming trend is punctuated by several short-term oscillations of different amplitude and duration (Fig. 2). From the Roman Period to 1700 CE, the SST shows a turnover into a cooling trend of 4.5 °C ± 2.1 °C (Fig. 2). The SST record ends from 1700 to 2014 CE with a short warming trend (Fig. 2).

**Figure 3:**
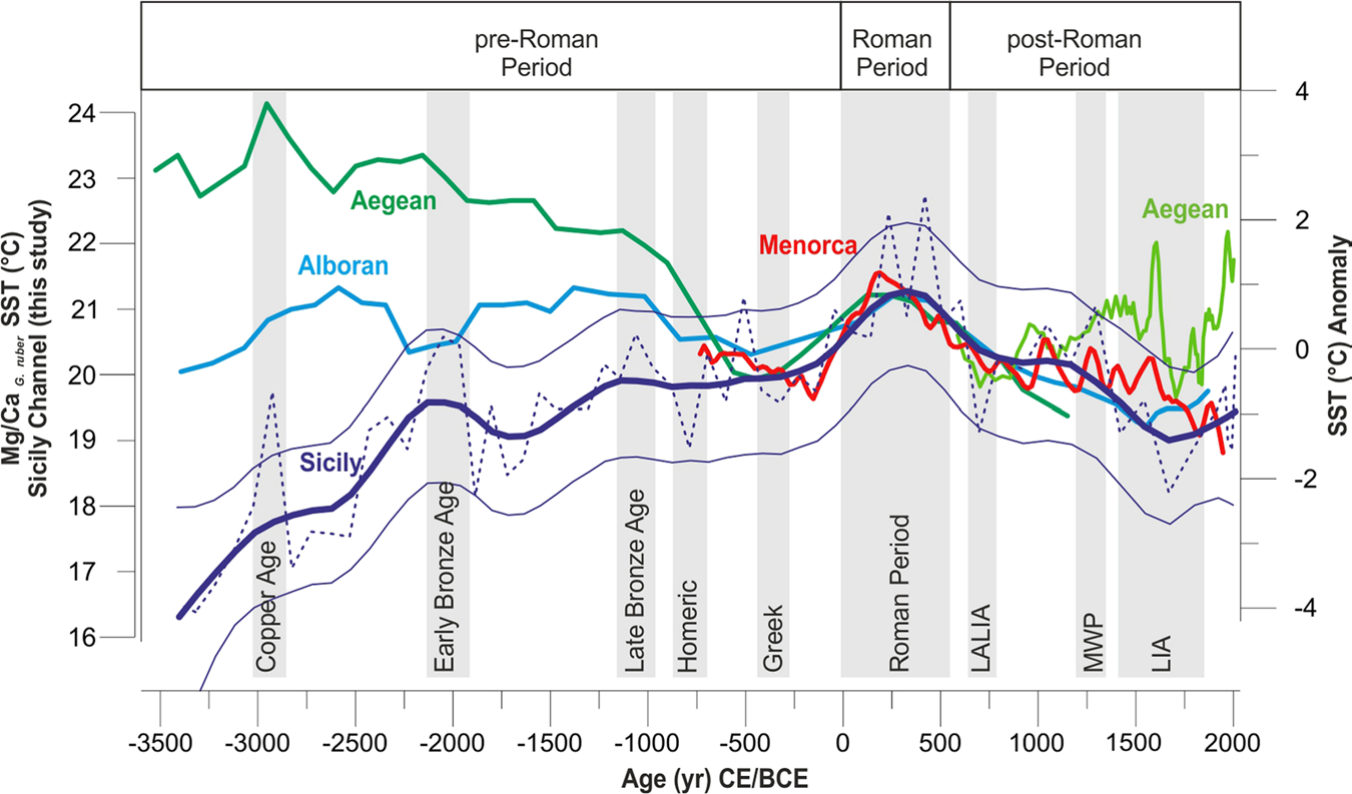
Comparison of the SSTs records from Sicily Channel (thick dark blue line, Mg/Ca _*G. ruber*_ core SW104-ND11, this work), Alboran Sea^39^ (thick light blue line), Minorca Basin^16^ (thick red line) and Aegean Sea^40,41^ (thick dark and light green lines) expressed as SST anomalies in relation with the reference period from 750 BCE to 1250 CE (the only period shared by all the records) in order to better compare the amplitude of the changes across the Mediterranean. The Sicily Channel SST raw data (dark blue dashed line, this work) are superimposed on the 95% CI smoothed curve computed as the 2.5% (dark blue thin line) and 97.5% (dark blue thin line) quantile of the 10000 smoothed values (see Material and Methods paragraph). The SST (°C) graduated scale is referred only to the Sicily Channel SST raw data. The Alboran Sea (thick light blue line), Minorca Basin (thick red line) and Aegean Sea (thick dark and light green lines) SST records are represented as 3 points running average. The grey bands show the main climate events documented in the Mediterranean basin and discussed in the text (e.g. Roman Period^62^).

This record is compared with other previously published SST reconstructions from the Mediterranean Sea (Fig. 2): an alkenone-SST record from the Alboran Sea^39^, a Mg/Ca_*G.bulloides*_ SST-stack record that integrates five Mg/Ca-SST records from the North Balearic Islands^16^ and a composite of two alkenone-SST records from the Aegean Sea^40,41^. This comparison is complemented with a record of north Hemisphere temperature anomalies^42^ and a reconstructed NAO record^28^. Thus, the compared SST records involve different proxies and calibrations with their own uncertainties. In order to facilitate their comparison, and eliminating potential biases in the absolute SST reconstruction associated with the applied calibrations or methods, we decided to compare the SST records transferring them into SST anomaly records in relation to a common reference period. Considering that the time interval covered by the considered marine records was different, the temperature anomalies (°C) were calculated respect to the only period shared by all the records, from 750 BCE to 1250 CE (Fig. 3).

Although some other Mediterranean SST records exist for the last millennia, previous compilation efforts have shown significant discrepancies in the main trends among different regions^11,43^. In this exercise, we have focussed on those records that are not directly influenced by large river outflows, human impact and local oceanographic circulation that could potentially alter the wide regional signal (See the supplementary material for detailed arguments on the criteria for record selection, paragraph S2). Different proxies reflect different seasons and depth habitats and thus SST signals are not expected to be identical among the chosen records. Nevertheless, this comparison exercise focuses on the main patterns and aims to evaluate their regional perseverance. In this regard, the most perseverant feature among the compared records is the well developed SST maximum recorded during the Roman Period (1–500 CE) (Fig. 3). The discussion on the SST evolution previous and posterior to this period is mostly focussed in the new Sicily SST record here presented.

### Pre-roman period

The comparison of the Mediterranean SST records evidences very distinct regional patterns before the onset of the Greek period, with an overall cooling trend in the Aegean Sea, a warming trend in the Sicily Strait and more stable conditions in the Alboran Sea (Fig. 2). In the Sicily Channel, between the base of the record and the beginning of the Bronze Age period, the Mg/Ca_*G.ruber*_ SST signal shows two warm events, at ca. 2913 BCE and ca. 2040 BCE, respectively (Fig. 2). The first warm event, documented in the study record of Sicily Channel, although it does not fall within the 95% CI (Fig. 2), has been tentatively associated to the Copper Age warm phase (ca. 2913 BCE). This event chronologically corresponds to a warming in the Aegean Sea SST record (Fig. 2) and agrees to the beginning of a gradual aridification process described in northern Egypt^44^.

The second one (Early Bronze Age, ca. 2040 BCE, Fig. 2) is associated with a further aridity phase, as documented by the strong decrease in arboreal pollen in the central Mediterranean^45,46^. This latter event, chronologically corresponds to the fall of the ancient Egyptian Reign^44^ and to the end of the Mesopotamian civilization, associated with strong famines related to a dry climate and a strong aridity^47^. Later, Mg/Ca _*G.ruber*_ SST warmed between 1800-1100 BCE (Fig. 2) indicating relative warm conditions during Late Bronze Age (ca. 1100 BCE) documented in the whole Mediterranean basin.

Significant cultural changes are documented during this period, in fact, historical records indicate the collapsed several civilizations^48,49^. Most of the Greek Bronze Age Palatial centres were destroyed and/or abandoned^50^. The Palatial centers were hit hard by the increase in aridity and the collapse of agriculture production^48^ making impossible for the population to sustain themselves^50,51^. The transition from Bronze Age to Iron Age chronologically approximate the short-term cooling event associated to the Homeric (ca. 800 BCE) grand solar minimum (Fig. 2). During this period, associated to negative NAO values, the climate condition were favourable to agriculture expansion in the eastern Mediterranean^44^. Compared to the subsequent Roman period, the Mediterranean was characterized by a colder phase from ca. 500 BCE to 200 BCE (Fig. 2), predating the rise of the Roman Empire. From a chronological point of view, this interval corresponds to the beginning of the so-called “sub-Atlantic phase”^47,52,53^, characterized by a cool climate and rainy winters which was propitious for the expansion of the Greek, Etruscan and Roman civilizations^54^. During this period, also global glacier advances are documented^55^ and a negative NAO phase is recorded (Fig. 2). The cool and humid climate of the sub-Atlantic phase lasted until ca. 100 BCE and covered the entire period of the Monarchy in Rome^47^. In addition, this period is characterised by a short-term cooling event associated with the Greek (ca. 350 BCE) solar minimum (Fig. 2).

However, at ca. 400 BCE, cultural changes were synchronised across the Mediterranean region^14^. The Greek and Phoenician colonies expanded and Rome and Carthage began their epic rise^14^, a situation coincident with the establishment of more homogeneous temperature conditions across the Mediterranean regions.

### Roman period

All the considered SST reconstructions, from the Alboran basin to the Aegean Sea, show homogeneous climate conditions at regional scale with the occurrence of a distinct warming phase from 1 CE to 500 CE (Fig. 3), coincident with the Roman Period and covering the whole Roman Empire archaeological period. This pronounced warming during the Roman Period is almost consistent with other marine records from Atlantic Ocean^56–58^ and with the continental anomaly reconstruction from Europe from PAGES 2 K, 2013^59^. This climate phase corresponds to the so-called “Roman Climatic Optimum” characterized by prosperity and expansion of the empire.

The described warm climate conditions of the “Roman Climatic Optimum” appear independently of the considered SST proxies. In one side, Mg/Ca_*G. ruber*_ SST are expected to reflect temperatures from late summer to autumn^60^ while of Mg/Ca_*G. bulloides*_ SST in the Menorca area has been interpreted to reflect spring SST conditions^16^. On the other side, alkenone-SST signal is interpreted as mean annual averages^12,16,39,61^. The good agreement between the different SST proxies and Mediterranean regions reflecting warm conditions during the whole Roman Period suggests warm climate conditions during the whole annual cycle and at regional level. Nevertheless, some differences in the intensity of the warming arise between the different proxy records. This warming in SST reconstructions range from 19.6 ± 1.5 °C to 22.7 ± 1.5 °C in the Mg/Ca_*G. ruber*_ SST of the Sicily Channel (Fig. 2), from 16.6 to 18.5 °C in the Mg/Ca_*G. bulloides*_ SST from the Menorca basin record^16^ and from 14.4 to 16.1 °C in the U^k^_37_ SST anomaly from both the Alboran and Aegean seas^39,40^. The overall larger Holocene variability in Mg/Ca-SST records in relation to alkenone-SST records have been previously recognized in the Alboran Sea and discussed to reflect the rather averaged and smoothed annual SST signal of the alkenone measurements^61^. Thus, our inter-basin comparison suggest that the warming was either more perseverant during the summer months or it was stronger in the central part of the Mediterranean in contrast to the eastern and western basins. The warmest part (ca. 250–420 CE) of the Mediterranean Roman Period is almost coincident with the warm phase in the north Hemisphere temperature anomalies^42^ (Fig. 2), suggesting a teleconnection between marine and continental system. Indeed, the “Roman Climatic Optimum” reveals itself as a phase of warm stable temperatures across much of the Mediterranean heartland of empire and covers the whole phase of origin, expansion and decline of the Roman Empire (Roman Climate Optimum, Roman Transitional Period and Late Antique Little Ice Age, according to Harper, 2019)^62^. Nevertheless, the expansion of the Roman Empire did not occur synchronously throughout the Mediterranean area because of the political situation, geographical pattern and, probably, also the specific climatic conditions in the various areas. However, starting from ca. 50 CE, the Roman Empire began its ascent towards north of Alps and the conquest of Gaul, probably assisted by the favourable climatic conditions that allowed this great feat. But, a shift from negative to positive NAO index values (Fig. 2) during the Roman period suggests a transfer of North Atlantic precipitation towards the central and northern Europe. In fact, positive NAO could indicate drier conditions by the second half of the Roman Period penalising southern Europe and North Africa economic development (i.e. agriculture)^63,64^. This feature agrees with the new finding by recent studies on vegetation patterns^46,65^ about the opening of vegetation in central/eastern Mediterranean areas since the ca. 100 CE. Thereafter the Rome expansion in central Europe and Great Britain was inexorable.

Therefore, it is possible to hypothesize that the auspicious climatic conditions of this period, the so-called “Roman Climatic Optimum”, may have contributed to the expansion of the Roman Empire in the various areas of the Mediterranean Sea probably linked to new needs and opportunities. By the end of this period, all the Mediterranean SST records show a generally consistent SST cooling trend (Fig. 3). During this period, both the Aegean Sea and Sicily Channel were laid siege by pirates and vandals. In fact, at the end of the IV century, the roman world was experiencing the beginning of the end that will culminate just few centuries later^66^. Sicily was a major supplier of grain throughout the Empire, and held the greatest part of the agrarian economy^67^. The island, deprived of military defences, was besieged by a band of pirates Illyrian (438 CE) which devastated a large stretch. In the same time, the Vandals of Genserico (440–477 CE) invaded the Eastern part of the Mediterranean Sea^67^.

### Post roman period

The post-Roman Period was characterised by a progressive cooling trend of 4.5 °C ± 2.1 °C that ended by the Little Ice Age (LIA) interval (Fig. 2) and was punctuated by two short-term events well-known as the Late Antique Little Ice Age (LALIA) and the Medieval Warm Period (MWP). The LALIA cooling event^23^, at ca. 650–700 CE (Fig. 2), has been recently documented in marine records of the western Mediterranean basin^9,10^ and corresponds to a period characterised by a decrease in alboreal pollen index in the south-central Mediterranean, suggesting the occurrence of cold and dry climate condition^45,46^. The LALIA event had an additional environmental stress led by the establishment of the Justinian plague while the eastern Roman Empire transformed, the Sasanian Empire collapsed, and population movements occurred out of the Asian steppe and Arabian Peninsula, which spread Slavic-speaking peoples and political upheavals in China^23^. Instead, the MWP, dated at ca. 1300 CE (Fig. 2) and characterised by overall warmer SST than the LALIA event, is considered by several authors^7,9,22,68–70^ as a relatively stable and warm period. Recently, Margaritelli *et al*.^10^ documented the occurrence of this warm event at Mediterranean regional scale.

The cooling of 2.8 °C ± 2 °C associated to the LIA event occurring between ca. 1300 CE and ca. 1700 CE affects the whole Mediterranean area (Fig. 2). This event is also well documented in several marine records of the Mediterranean^5–10,16,71–73^. After 1700 CE, the SST from Sicily Channel, tend to warm (Fig. 2) although interrupted by a short cooling at 1980 CE^74^. This warming phase fits with the Aegean Sea SST record and with the north Hemisphere temperature anomalies^5,40,75,76^. This feature is probably associated to the onset of the Industrial Period/Modern Warm Period.

## Conclusions

The new generated Mg/Ca_*G. ruber*_ SST record from Sicily channel allows to identify a series of climate events that can be associated to different remarkable socio-cultural developments of ancient Mediterranean civilizations for the last five millennia. Warm events are associated to historical periods such as the Cooper age, the Early and Late Bonze age while cold events are associated to the Homeric and Greek periods. The comparison of this new SST record with previous published Mediterranean SST records from Alboran Sea, Minorca Basin and Aegean Sea, highlight the overall perseverance of warm conditions during the Roman Period (1 CE to 500 CE). These warm conditions were particularly intense in the Sicily record, that reflect summer months, and correspond to the so called “Roman Climatic Optimum”. During this period, in fact, developed the greatest ancient civilization of all time, the Roman one. We hypothesise the relevance that these climate conditions may had in the expansion of the Roman Empire and its collapse with the general development of colder conditions. A cooling trend dominated after the Roman period reaching minimum values, in the whole Mediterranean, by the end of the LIA, minor oscillations punctuated this cold trend and were also associated with socio-cultural changes in central Mediterranean region.

## Materials and methods

### Core description

Core SW104-ND11 (37°01′57″N, 13°10′54″E - water depth of 475 m) is located in the northwestern part of the Sicily Channel (Fig. 1) and was recovered during NEXTDATA 2014 oceanographic expedition on board the R/V CNR-Urania. The sedimentary sequence has been recovered using the SW104 gravity corer system, which allows the recovery of undisturbed and very well preserved water-sediment interface. The core length is of 119 cm and consists of homogeneous grey hemipelagic sediments.

### Mg/Ca-SST

About 60 selected individuals of *Globigerinoides ruber* white variety *sensu scricto* (>125 µm) were used per sample. The tests were crushed under glass slides to open the chambers and carefully cleaned applying a sequence of clay removal, reductive, oxidative and weak acid cleaning steps^77^. A preliminary cleaning test was performed in order to adjust the cleaning protocol to the sample characteristics. In base to the Al/Ca and Mn/Ca ratios in the samples and also in the residuals of the cleaning steps it was decided that the reductive step was needed (Fig. S1a) but also additional clay removal steps were added in order to reduce the relatively high Al values. Additionally, the appropriate performance of the chosen cleaning protocol was further tested by visual inspection of the interior of the foraminifera walls by SEM-microscopy (Fig. S1b) in some chosen samples previously and after their cleaning. These SEM pictures (Fig. S1b) show an efficient removal of diagenetic and detritic carbonates attached to the foraminifera wall after the application of the described full cleaning protocol. Procedural blanks were routinely measured to discard any potential contamination problem during cleaning and dissolution. Instrumental analyses were performed in an inductively coupled plasma mass spectrometer (ICP-MS Perkin Elmer ELAN 6000) in the Scientific and Technological Centers of the University of Barcelona (CCiT-UB). A standard solution with known elemental ratios was used for sample standard bracketing (SSB) as a correction for instrumental drift. The Mn/Ca and Al/Ca ratios were always measured to identify potential contaminations due to the presence of manganese oxides and/or aluminosilicates^77–80^ (Fig. S1a). Measured samples always show Mn/Ca and Al/Ca values below 0.1 mmol/mol and 0.4 mmol/mol respectively (thresholds defined by some previous studies^77,78,80^) (Fig. S1a). However, for this study those Mn/Ca ratios above 2σ (0.09 mmol/mol; over standard deviations of the averages Mn/Ca values) were removed. Also those Al/Ca ratios with values below 2σ (0.21 mmol/mol). Those values bellow 2σ have been accepted as a good result since any clear covariance with Mg/Ca ratios exist (Fig. S1a). The Mg/Ca ratios were converted in Sea Surface Temperature (SST) values according to the *G. ruber* calibration from Elderfield and Ganssen (2000)^81^. The SST error has been estimated after propagating the errors associated to the Mg/Ca ratio measurements and to the used SST calibration according to the procedure described in the supplementary material (S1). Therefore, the resulting propagated errors from the Core SW104-ND11 covers an error range between ±1.35 °C to ±1.54 °C.

The estimated core top Mg/Ca_*G.ruber*_ SST of the study record is 20 ± 1.4 °C and this temperature is consistent with the measured temperature at ca. 20 metres in July 2014 during the sampling (Fig. 1c). This value is also in agreement with the seasonal temperature distribution at ca. 20 metres in the Sicily Channel evaluated with the World Ocean Atlas 2013^82^ dataset that averages measurements from 1955 to 2012. Even the living *G. ruber* data from Pujol and Vergnaud Grazzini (1995)^59^ and Mallo *et al*.^83^ support the depth habitat of *G. ruber* in the Sicily Channel at ca. 20 metres and 20 °C.

Some previous studies have argued that Mg/Ca ratios in the Mediterranean were anomalously high due to the effect of high salinities in the region^84^. However, later studies indicate that such a high Mg/Ca ratios in warm oversaturated waters resulted from the chemical overprint of attached secondary calcite^85,86^. These observations are also in agreement with later culture experiments that reported relatively low sensitivity of the Mg/Ca ratio to high salinity conditions^87^. This study has taken particular care in ensuring the removal of any potential digenetic calcite by applying the so-called reductive cleaning step, which has demonstrated to promote the release of secondary calcite overgrowths^77,80^. The efficiency of the cleaning protocol has further been checked through the visual inspection of the cleaned foraminifera walls by SEM-microscopy (Fig. S1b). In addition, the obtained Mg/Ca ratios in the core top provide coherent SST for the late summer season when *G. ruber* growths in the region^60^. They are also coherent with the *G. ruber*-Mg/Ca values previously reported in the same region for a record covering the last 5 centuries^76^. Therefore, we can conclude that our Mg/Ca_*G.ruber*_ represents a reliable SST record with no major interferences of diagenetic artefacts in the signal.

### Chronology

The chronology for the uppermost 10 cmbsf (cm below seafloor) of core SW104-ND11 is estimated by radionuclides ^210^Pb and ^137^Cs. The ^210^Pb and ^137^Cs analysis were carried out at ISMAR-CNR Bologna, according to the procedures reported in Bellucci *et al*.^88^. The ^137^Cs activity-profile does not show a clear peak corresponding to 1963 CE (maximum ^137^Cs fallout from nuclear testing) in the first few cmbsf, suggesting the presence of a surficial mixed layer from 0 to 7 cmbsf. Considering that ^137^Cs from tests of nuclear weapons was first detected in the atmosphere in 1954 CE, we assigned this age at 7 cmbsf, where this radionuclide first appears (Fig. S2). Using the ^210^Pb profile below the mixed layer and applying the CF–CS model (Constant Flux–Constant Sedimentation), we calculate a mean sedimentation rate of 50 yr/cm.

The four AMS ^14^C radiocarbon analysis on mixed planktonic foraminifera (Tab. S1) were performed at iCONa Lab of the Dipartimento di Scienze e Tecnologie Ambientali dell’Università della Campania and at the laboratory LABEC (Laboratorio di tecniche nucleari per l’Ambiente e i Beni Culturali) of the INFN - Florence. Radiocarbon ages were calibrated using the MARINE13 calibration curves^89^. The calibrated age ranges are reported in years CE and refer to 2σ.

The age model for core SW104_ND11 was constructed using the Bayesian statistics software Bacon with the statistical package R^90,91^ for marine sediments using the four AMS ^14^C radiocarbon ages and the tie-point associated to the first detection of ^137^Cs in the study core. This age model shows a near-constant sedimentation rate with a mean value of 50 yr/cm from the top down to the base of the study core (Fig. S3).

### Regression analysis of temperatures temporal profile

In order to statistically validate the observed trends in temporal profile, temperature values were smoothed by adopting a non-parametric regression approach. The adopted procedure was similar to the one applied in Martínez-Boti *et al*.^92^ and allow to validate the observed trend by taking into account all sources of uncertainty in temperature values while maintaining fixed the age model. Specifically, a LOESS (LOcally Estimated Scatterplot Smoothing^93^) fitting was carried out. LOESS belongs to the family of non-parametric fitting procedure and does not require the a priori specification of the relationship between dependent variable and predictors. In LOESS regression the user must specify the so called “span” (or “alpha”) parameter, determining the proportion of observation used in each local regression. The span parameter, ranging between 0 and 1, strongly influence the regression results; higher span values tends to produce too much smoothed curves, thus hiding the informative part of the regression, while lower span values will produce too much wiggling curves thus fitting the noise rather than the dominant signal. In order to identify the optimal span parameter, a v-fold cross validation and a generalized cross-validation (GCV) methods were used. Optimal span values were 0.17 and 0.19 as inferred by v-fold cross-validation and GCV respectively, thus a span value of 0.18 was used in LOESS regression. Finally, once the optimal span values was determined, a Monte Carlo simulation was adopted to take into account all the possible source of variability. In particular, 10000 possible realization of the temperature time series were generated by randomly sampling each Mg/Ca observation within its uncertainty bound (2σ), and computing the new temperature value according to Elderfield and Ganssen^81^ equation:_Mg/Ca(mmol·mol-1)=0.52exp0.1 T. The LOESS regression was then carried out for each realization and for each data-point the smoothed value was computed as the mean value of the 10000 smoothed values. Accordingly, the 95% CI for the smoothed curve were computed as the 2.5% and 97.5% quantile of the 10000 smoothed values. All the calculations were carried out in R statistical environment (R Core Team, 2019) by using packages fANCOVA^94^ and paleoMAS^95^ to perform generalized cross-validation and v-fold cross validation respectively. However, it is important to state that the discussions and interpretations of SST variability documented in the identified climate events (in the study core SW1044-ND11, Sicily Channel) refer to the measured data points in °C; the LOESS regression and its CI obtained by using Monte Carlo simulation in fact, has been used only to validate the main trends observed in the SST data.

## Supplementary information

Supplementary information.

## Data Availability

The data from Alboran Sea (Rodrigo-Gamiz *et al*.^39^), Minorca Basin (Cisneros *et al*.^16^), from Aegean Sea (Kontakiotis, 2016; Gogou *et al*.^40,41^) and the north Hemisphere temperature reconstruction (Ljungqvist *et al*.^42^) were recovered from the original publications. The Mg/Ca_*G.ruber*_ data from the Sicily Channel are available on Pangaea data publisher.

## References

[1] Giorgi F (2006). Climate change hot-spots. Geophysical Research Letters.

[2] Corte-Real J, Zhang X, Wang X (1995). Downscaling GCM information to regional scales: A non-parametric multivariate regression approach. Climate Dynamics.

[3] Xoplaki E. Climate Variability over the Mediterranean. Ph.D. Thesis, University of Bern, 193 pp. Available at, http://sinus.unibe.ch/klimet/docs/phd_xoplaki.pdf (2002).

[4] Lionello, P., Malanotte-Rizzoli, P. & Boscolo, R. (Eds). Mediterranean Climate Variability. Elsevier, pp 438. ISBN: 0-444-52170-4, (2006).

[5] Grauel AL, Goudeau MLS, de Lange GJ, Bernasconi SM (2013). Climate of the past 2500 years in the Gulf of Taranto, central Mediterranean Sea: a high-resolution climate reconstruction based on δ18O and δ13C of Globigerinoides ruber (white). The Holocene.

[6] Goudeau MLS (2015). Seasonality variations in the Central Mediterranean during climate change events in the Late Holocene. Palaeogeography, Palaeoclimatology, Palaeoecology.

[7] Lirer F (2014). Planktonic foraminifera as bio-indicators for monitoring the climatic changes that have occurred over the past 2000 years in the southeastern Tyrrhenian Sea. Integrative Zoology.

[8] Bonomo S (2016). Reworked Coccoliths as runoff proxy for the last 400 years: The case of Gaeta Gulf (central Tyrrhenian Sea, Central Italy). Palaeogeography, Palaeoclimatology, Palaeoecology.

[9] Margaritelli G (2016). Marine response to climate changes during the last five millennia in the central Mediterranean Sea. Global and Planetary Change.

[10] Margaritelli G (2018). Climatic variability over the last 3000 years in the central – western Mediterranean Sea (Menorca Basin) detected by planktonic foraminifera and stable isotope records. Global and Planetary Change.

[11] Jalali B, Sicre MA, Bassetti MA, Kallel N (2016). Holocene climate variability in the North-western Mediterranean Sea (Gulf of Lions). Climate of the Past.

[12] Jalali, B. et al. High-resolution Holocene climate and hydrological variability from two major Mediterranean deltas (Nile and Rhone). The Holocene 1–11 (2017).

[13] Labuhn, I., Finné, M., Izdebski, A., Roberts, N., Woodbridge, J. Climatic Changes and Their Impacts in the Mediterranean during the First Millennium AD. Environment and Society in the Long Late Antiquity, (Late Antique Archaeology 12), (Leiden 2018), Adam Izdebski and Michael Mulryan (eds), 65–88 (2018).

[14] Roberts N, Brayshaw D, Kuzucuoglu C, Perez R, Sadori L (2011). The mid-Holocene climatic transition in the Mediterranean: Causes and consequences. The Holocene.

[15] McCormick M (2012). Climate Change during and after the Roman Empire: Reconstructing the Past from Scientific and Historical Evidence. Journal of Interdisciplinary History.

[16] Cisneros M (2016). Sea surface temperature variability in the central-western Mediterranean Sea during the last 2700 years: a multi-proxy and multi-record approach. Climate of the Past.

[17] Neukom, R., Steiger, N., Gómez-Navarro, J.J., Wang, J., Werner, J.P. No evidence for globally coherent warm and cold periods over the preindustrial Common Era. Nature 571 (2019). 10.1038/s41586-019-1401-2 31341300

[18] Neukom R (2019). Consistent multidecadal variability in global temperature reconstructions and simulations over the Common Era. PAGES 2k Consortium. Nature Geosciences.

[19] Magny M, Combourieu Nebout N (2013). Holocene changes in environment and climate in the central Mediterranean as reflected by lake and marine records. Climate of the Past.

[20] Holmgren K (2016). Mediterranean Holocene climate, environment and human societies. Quaternary Science Reviews.

[21] Sadori L (2016). Climate, environment and society in southern Italy during the last 2000 years. A review of the environmental, historical and archaeological evidence. Quaternary Science Reviews.

[22] Büntgen, U. & Tegel, W. European tree-ring data and the Medieval Climate Anomaly. PAGES 19, 14–15 (2011).

[23] Büntgen U (2016). Cooling and societal change during the Late Antique Little Ice Age from 536 to around 660 AD. Nature Geoscience.

[24] Hodell D, Brenner M, Curtis JH, Guilderson T (2001). Solar forcing of drought frequency in the Maya lowlands. Science.

[25] deMenocal PB, Peter B (2001). Cultural Responses to Climate Change During the Late Holocene. Science.

[26] IPCC, 2018: Summary for Policymakers. In: Global warming of 1.5 °C. An IPCC Special Report on the impacts of global warming of 1.5 °C above pre-industrial levels and related global greenhouse gas emission pathways, in the context of strengthening the global response to the threat of climate change, sustainable development, and efforts to eradicate poverty. World Meteorological Organization, Geneva, Switzerland, 32, V. Masson-Delmotte, et al. (eds.) (2018).

[27] Fischer H (2018). Palaeoclimate constraints on the impact of 2 °C anthropogenic warming and beyond. Nature Geoscience.

[28] Faust JC, Fabian K, Milzerc G, Giraudeau J, Kniesa J (2016). Norwegian fjord sediments reveal NAO related winter temperature and precipitation changes of the past 2800 years. Earth and Planetary Science Letters.

[29] Pinardi N (2015). Mediterranean Sea large-scale low-frequency ocean variability and water mass formation rates from 1987 to 2007: A retrospective analysis. Progress in Oceanography.

[30] La Violette PE (1990). The western Mediterranean circulation experiment (WMCE): introduction. Journal of Geophysical Research.

[31] Viudez A, Tintore J, Haney RL (1996). Circulation in the Alboran Sea as determined by quasi-synoptic hydrographic observations. Part 1. Three-dimensional structures of the two anticyclonic gyres. Journal of Physical Oceanography.

[32] Viudez A, Tintore J (1995). Time and space variability in the eastern Alboran Sea from March to May 1990. Journal of Geophysical Research.

[33] Millot C (1987). Circulation in the western Mediterranean Sea. Oceanologica Acta.

[34] Krom M.D., Groom S. & Zohary T. The Eastern Mediterranean — Biogeochemistry of Marine Systems. Blackwell Publishing (2003).

[35] Bethoux J-P (1980). Mean water fluxes across sections in the Mediterranean Sea, evaluated on the basis of water and salt budgets and of observed salinities. Oceanologica Acta 3.

[36] Robinson AR (1999). The Atlantic Ionian Stream. Journal of Marine Systems.

[37] Bèranger K (2004). The dynamics of the Sicily Strait: A comprehensive study from observations and models. Deep-Sea Research Part II.

[38] Incarbona A (2008). Holocene millennial-scale productivity variations in the Sicily Channel (Mediterranean Sea). Paleoceanography.

[39] Rodrigo-Gámiz M, Martínez-Ruiz F, Rampen SW, Schouten S, Sinninghe Damsté JS (2014). Sea surface Temperature variations in the western Mediterranean Sea over the last 20 kyr: A dual-organic proxy (UK′37 and LDI) approach. Paleoceanography.

[40] Kontakiotis G (2016). Late Quaternary paleoenvironmental reconstruction and paleoclimatic implications of the Aegean Sea (eastern Mediterranean) based on paleoceanographic indexes and stable isotopes. Quaternary International.

[41] Gogou A (2016). Climate variability and socio-environmental changes in the northern Aegean (NE Mediterranean) during the last 1500 years. Quaternary Science Reviews.

[42] Ljungqvist FC (2010). A new reconstruction of temperature variability in the extra-tropical Northern Hemisphere during the last two millennia. Geografiska Annaler Series A: Physical Geography.

[43] Jalali B (2018). Deltaic and coastal sediments as recorders of Mediterranean regional climate and human impact over the past three millennia. Paleoceanography and Paleoclimatology.

[44] Kaniewski D (2010). Late Second-Early First Millennium BC abrupt climate changes in coastal Syria and their possible significance for the history of the Eastern Mediterranean. Quaternary Research.

[45] Di Rita F (2018). Late Holocene forest dynamics in the Gulf of Gaeta (central Mediterranean) in relation to NAO variability and human impact. Quaternary Science Reviews.

[46] Di Rita F (2018). Holocene forest dynamics in central and western Mediterranean: periodicity, spatio-temporal patterns and climate influence. Scientific Reports.

[47] Behringer, W. A Cultural History of Climate. London, Polity Press, ISBN: 9780745645292, (2009).

[48] Weiss B (1982). The decline of the Late Bronze Age civilization as a possible response to climate change. Climatic Change.

[49] Knapp B, Manning SW (2016). Crisis in Context: The End of the Late Bronze Age in the Eastern Mediterranean. American Journal of Archaeology.

[50] Drake BL (2012). The influence of climatic change on the Late Bronze Age Collapse and the Greek Dark Ages. Journal of Archaeological Science.

[51] Finnè M (2017). Late Bronze Age climate change and the destruction of the Mycenaean Palace of Nestor at Pylos. PLoS ONE.

[52] Zolitschka B, Behre KE, Schneider J (2003). Human and climate impact on the environmental as derived from colluvial, fluvial and lacustrine archives-examples from the Bronze Age to the Migration period, Germany. Quaternary Science Reviews.

[53] Kotthoff U (2017). Reconstructing Holocene temperature and salinity variations in the western Baltic Sea region: a multi-proxy comparison from the Little Belt (IODP Expedition 347, Site M0059). Biogeosciences.

[54] Shaw, B.D. Climate, Environment, and History: the Case of Roman North Africa. Chap. 16 [in] T. M. L. Wigley, M. Ingram, and G. Farmer eds., Climate and History: Studies in Past Climates and their Impact on Man. Cambridge University Press, pp. 379-403 (1981).

[55] Mayewski PA (2004). Holocene climate variability. Quaternary Research.

[56] Bond G (2001). Persistent solar influence on North Atlantic climate during the Holocene. Science.

[57] Sicre MA (2008). A 4500- year reconstruction of sea surface temperature variability at decadal time-scales off North Iceland. Quaternary Science Reviews.

[58] DeMenocal P, Ortiz J, Guilderson T, Sarnthein M (2000). Coherent high- and lowlatitude climate variability during the Holocene warm period. Science.

[59] PAGES 2K Consortium (2013). Continental-scale temperature variability during the past two millennia. Nature.

[60] Pujol C, Vergnaud Grazzini C (1995). Distribution patterns of live planktic foraminifers as related to regional hydrography and productive systems of the Mediterranean Sea. Marine Micropaleontology.

[61] Català A, Cacho I, Frigola J, Pena LD, Lirer F (2019). Holocene hydrography evolution in the Alboran Sea: a multi-record and multi-proxy comparison. Climate of the Past.

[62] Harper, K. The Fate of Rome, Climate, Disease, and the End of an Empire. The Princeton History of the Ancient World Series. Princeton: Princeton University Press (2019).

[63] Noti R (2009). Mid- and late-holocene vegetation and fire history at Biviere di Gela, a coastal lake in southern Sicily, italy. Vegetation History and Archaeobotany.

[64] Bisculm M (2012). Holocene vegetation and fire dynamics in the supra-mediterranean belt of the Nebrodi Mountains (Sicily, Italy). Journal of Quaternary Science.

[65] Di Rita FD, Magri D (2012). An overview of the Holocene vegetation history from the central Mediterranean coasts. Journal of Mediterranean Earth Science.

[66] Gibbon, E. The History of the Decline and Fall of the Roman Empire. Strahan & Cadell, London (1776).

[67] Momigliano, A. La caduta senza rumore. Ed. scientifiche italiane, Italy (1973).

[68] Lamb, H.H. Climate: Present, Past and Future vol. 2. Methuen & Co, London (1977).

[69] Jones P, Mann D, Mann ME (2004). Climate over past millennia. Reviews of Geophysics.

[70] Mann ME (2009). Global signatures and dynamical origins of the Little Ice Age and Medieval Climate Anomaly. Science.

[71] Piva A, Asioli A, Trincardi F, Schneider RR, Vigliotti L (2008). Late Holocene climate variability in the Adriatic Sea (Central Mediterranean). The Holocene.

[72] Incarbona A (2010). The Impact of the Little Ice Age on Coccolithophores in the Central Mediterranea Sea. Climate of the Past.

[73] Sicre MA (2016). Sea surface temperature variability in the North Western Mediterranean Sea (Gulf of Lion) during the Common Era. Earth and Planetary Science Letters.

[74] Marullo S, Artale V, Santoleri R (2011). The SST Multidecadal Variability in the Atlantic–Mediterranean Region and Its Relation to AMO. Journal of Climate.

[75] Versteegh GJM, de Leeuw JW, Taricco C, Romero A (2007). Temperature and productivity influences on U37 K0 and their possible relation to solar forcing of the Mediterranean winter. Geochem. Geophys. Geosyst..

[76] Incarbona A (2016). Mediterranean circulation perturbations over the last five centuries: Relevance to past Eastern Mediterranean Transient-type events. Scientific Reports.

[77] Pena LD, Calvo E, Cacho I, Eggins S, Pelejero C (2005). Identification and removal of Mn-Mg-rich contaminant phases on foraminiferal tests: implications for Mg/Ca past temperature reconstructions. Geochemistry Geophysics Geosystems.

[78] Barker S, Cacho I, Benway H, Tachikawa K (2005). Planktonic foraminiferal Mg/Ca as a proxy for past oceanic temperatures: A methodological overview and data compilation for the Last Glacial Maximum. Quaternary Science Reviews.

[79] Lea DW, Pak DK, Spero HJ (2000). Climate impact of late Quaternary equatorial Pacific sea surface temperature variations. Science.

[80] Pena LD (2008). Characterization of contaminant phases in foraminifera carbonates by electron microprobe mapping. Geochemistry, Geophysics, Geosystems.

[81] Elderfield H, Ganssen G (2000). Past temperature and 18O of surface ocean waters inferred from foraminiferal Mg/Ca ratios. Nature.

[82] Boyer, T. & Mishonov, A. World Ocean Atlas 2013 Product Documentation (2013).

[83] Mallo M, Ziveri P, Mortyn PG, Schiebel R, Grelaud M (2017). Low planktic foraminiferal diversity and abundance observed in a spring 2013 west–east Mediterranean Sea plankton tow transect. Biogeosciences.

[84] Ferguson JE, Henderson GM, Kucera M, Rickaby REM (2008). Systematic change of foraminiferal Mg/Ca ratios across a strong salinity gradient. Earth and Planetary Science Letters.

[85] Hoogakker BAA, Klinkhammer GP, Elderfield H, Rohling EJ, Chris Hayward C (2009). Mg/Ca paleothermometry in high salinity environments. Earth and Planetary Science Letters.

[86] van Raden UJ, Groeneveld J, Raitzsch M, Kucera M (2011). Mg/Ca in the planktonic foraminifera Globorotalia inflata and Globigerinoides bulloides from Western Mediterranean plankton tow and core top samples. Marine Micropaleontology.

[87] Hönisch B (2013). The influence of salinity on Mg/Ca in planktic foraminifers – Evidence from cultures, core-top sediments and complementary d18O. Geochimica et Cosmochimica Acta.

[88] Bellucci LG (2007). 210Pb and 137Cs as chronometers for salt marsh accretion in the Venice Lagoon — links to flooding frequency and climate change. Journal of Environmental Radioactivity.

[89] Reimer PJ (2013). IntCal13 and Marine13 radiocarbon age calibration curves 0–50,000 years cal BP. Radiocarbon.

[90] Blaauw M, Christen JA (2011). Flexible Paleoclimate Age-Depth Models Using an Autoregressive Gamma Process. Bayesian. Analysis.

[91] R Core Team. R: A language and environment for statistical computing. R Foundation for Statistical Computing, Vienna, Austria., https://www.R-project.org/ (2019).

[92] Martínez-Boti MA (2015). Boron isotope evidence foroceanic carbon dioxide leakage during the last deglaciation. Nature.

[93] Cleveland, W.S., Grosse, E. & Shyu, W.M. Local regression models. Chapter 8 of Statistical Models in S eds J.M. Chambers and T.J. Hastie, Wadsworth & Brooks/Cole (1992).

[94] Wang, X,F. fANCOVA: Nonparametric Analysis of Covariance. R package version 0.5-1, https://CRAN.R-project.org/package=fANCOVA (2010).

[95] Correa-Metrio, A., Urrego, D.H., Kenneth, R. Cabrera and Mark B. Bush. paleoMAS: Paleoecological Analysis. R package version 2.0-1. (2012).

