# Supplementary material for: Persistent warm Mediterranean surface waters during the Roman period
Source: Sci Rep. 2020 Jun 26;10:10431. doi: 10.1038/s41598-020-67281-2 (PMC7319961; doi:10.1038/s41598-020-67281-2)
Supplement: Supplementary file 1 — Supplementary information. [file 41598_2020_67281_MOESM1_ESM.docx]

Persistent warm Mediterranean surface waters during the Roman period

Margaritelli G.^1,2*^, Cacho I.^2^, Català, A.^2^, Barra M. ^3^, Bellucci L.G.^4^, Lubritto C.^5^, Rettori R.^6^, Lirer F.^3^

1) Istituto di Ricerca per la Protezione idrogeologica (IRPI), CNR, via della Madonna Alta 126, 06128, Perugia, Italy

2) GRC Geociències Marines, Dept. de Dinàmica de la Terra i de l’Oceà, Facultat de Geologia, Universitat de Barcelona, Barcelona, Spain

3) Istituto di Scienze Marine (ISMAR), CNR, Calata Porta di Massa, Interno Porto di Napoli, 80133, Napoli, Italy

4) Istituto di Scienze Marine (ISMAR), CNR, Via Gobetti 101, 40129 Bologna, Italy

5) Dipartimento di Scienze e Tecnologie Ambientali Biologiche e Farmaceutiche (DiSTABiF), Università della Campania “Luigi Vanvitelli”, Via Vivaldi 47, Caserta, Italy

6) Dipartimento di Fisica e Geologia, Università degli Studi di Perugia, Via Alessandro Pascoli, 06123 Perugia, Italy


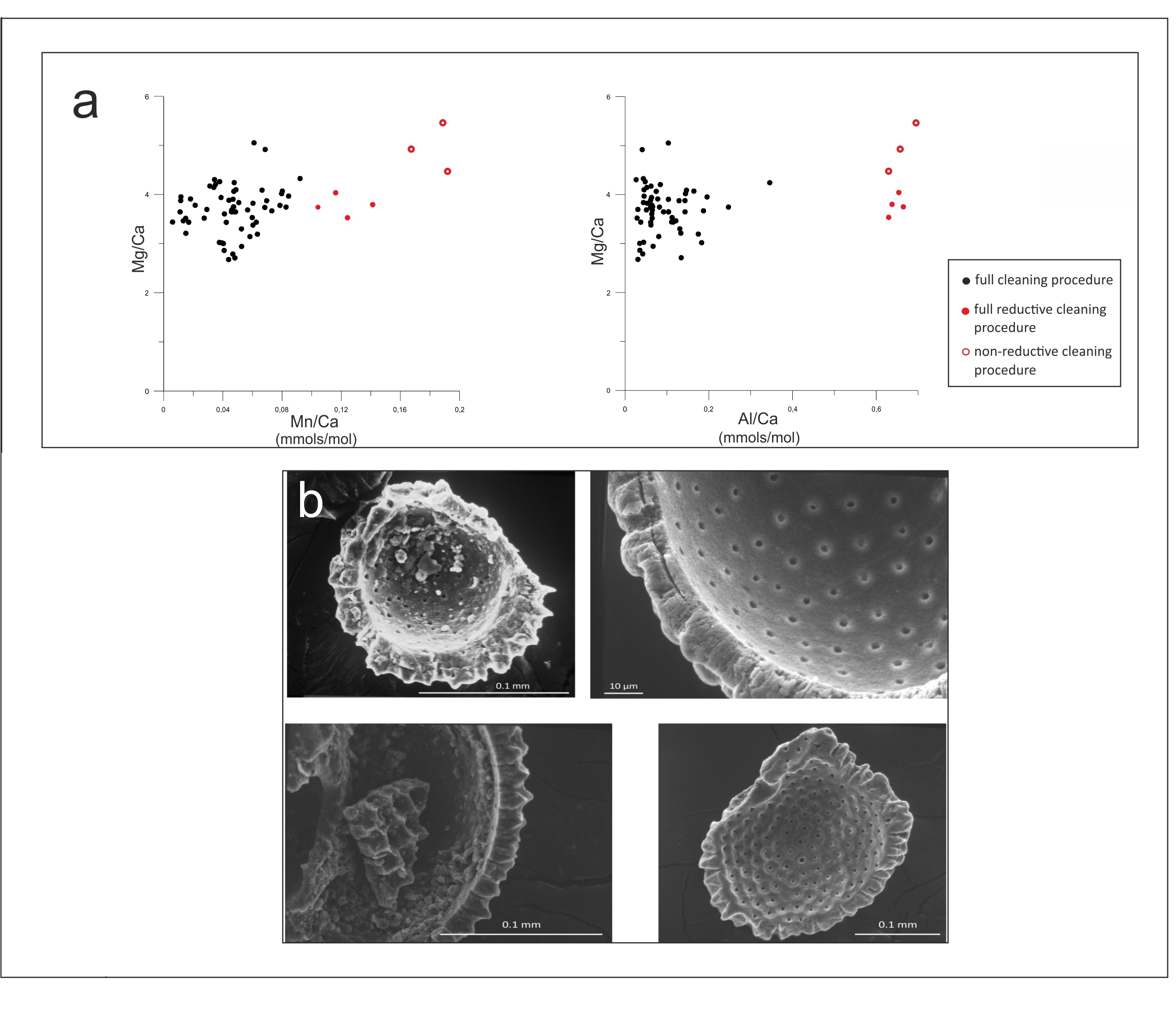


**Fig. S1:** **a)** Mn/Ca and Al/Ca ratios in relation to Mg/Ca ratios in mmol/mol measured in the *G. ruber* samples from core SW104-ND11. (open red dots) Cleaning test samples without the reductive cleaning step; (filled red dots) Cleaning test samples with reductive cleaning steps; (black dots) samples from the SW104-ND11 SST record cleaned with the reviewed protocol according to the text. **b)** SEM-microscopy pictures of *G. ruber* fragments showing the interior of the chambers before (left) and after (right) applying the chosen cleaning protocol. Note that fragments on the right show the efficient removal of attached secondary calcite or detritic material over the shell walls.


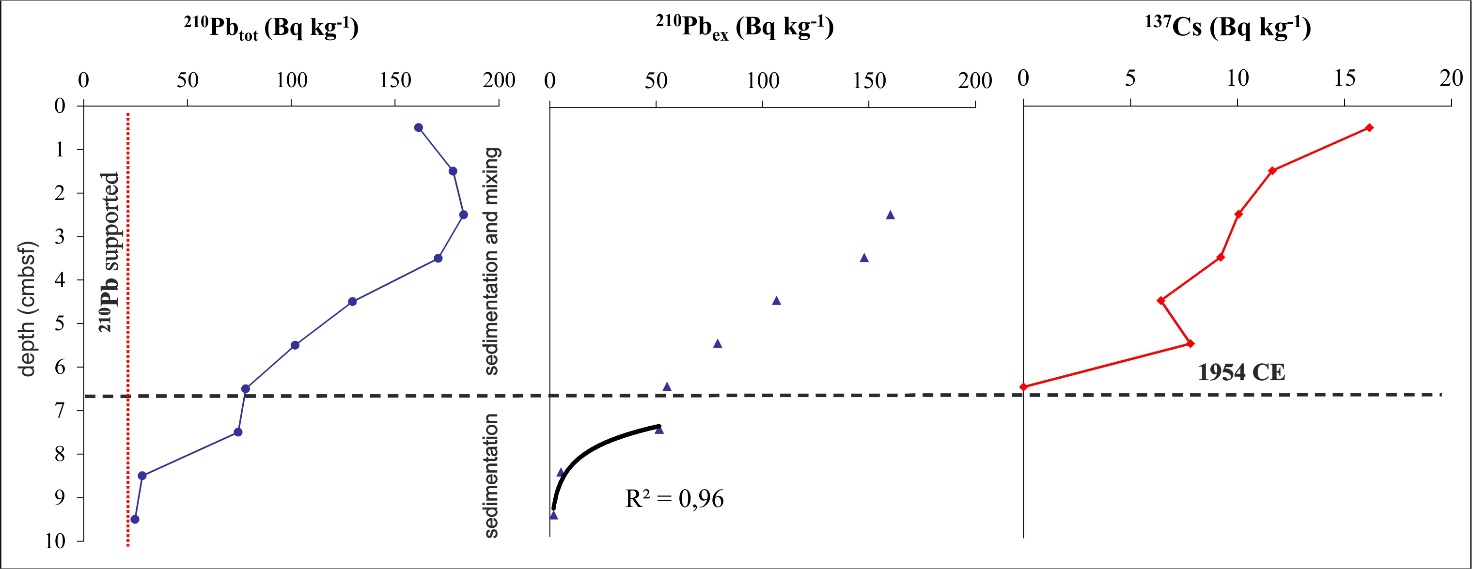


**Fig. S2:** The ^210^Pb and ^137^Cs activity-profiles documented in core SW104-ND11.


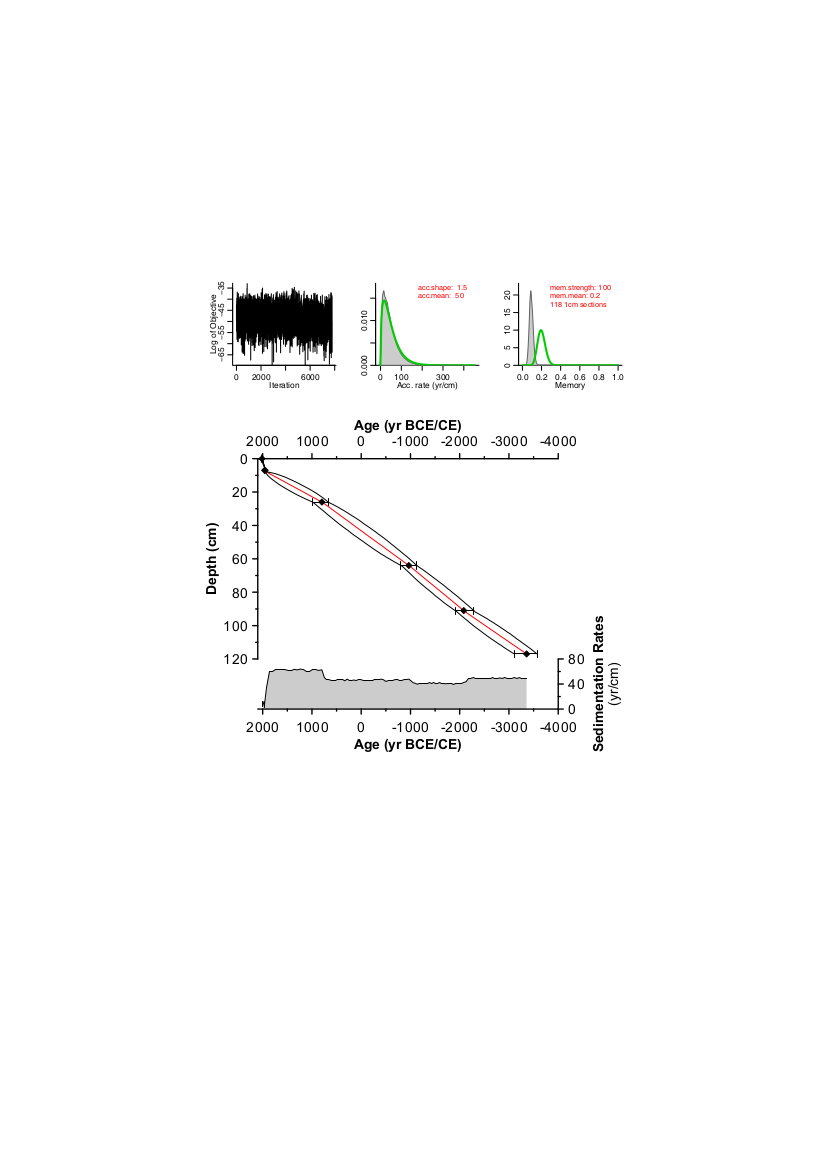


**Fig. S3:** Chronology for core SW104-ND11 after applying Bayesian statistics software Bacon with the calibrated 14C dates. The three upper graph depict the Markov chain Monte Carlo (MCMC) iterations. Left panel show a stationary distribution with little structures among neighbouring iterations. Green curves and grey histograms show the distribution for the accumulation rates (middle panel) and the memory or autocorrelation (right panel).

| **Core** | **Depth cm below sea floor** | **Sample** | **Lab. Code** | **Material** | **Radiocarbon Age** | **Age cal. Years BP** | **Age cal. Years CE/BCE** |
| --- | --- | --- | --- | --- | --- | --- | --- |
| SW104-ND11 | 25 | 25-26 | Fi 3273 | mixed planktonic  foraminifera | 1647 ± 46 | 1088 BP | 862 CE |
| SW104-ND11 | 63 | 63-64 | Fi 3276 | mixed planktonic  foraminifera | 3155 ± 48 | 2870 BP | 920 BCE |
| SW104-ND11 | 90 | 90-91 | Fi 3277 | mixed planktonic  foraminifera | 3971 ± 53 | 3992 BP | 2042 BCE |
| SW104-ND11 | 117 | 117 | Fi 3217 | mixed planktonic  foraminifera | 4970 ± 65 | 5309 BP | 3359 BCE |

**Tab.S1:** Information for the AMS ^14^C dated samples calibrated using the MARINE13 calibration curves. The calibrated age ranges are reported in years CE and refer to 2σ.

**S1. Error estimations in the sea surface record from core SW104-ND11**

The absolute uncertainty associated to each Mg/Ca ratio has been calculated considering the measurement precision (A) and the external reproducibility (B) as follows:

A) Measurement Relative Error of Mg/Ca ratios:

$$Mes.Rel. Err. \left( \frac{Mg}{Ca} \right)=\sqrt{\left( rsdMg \right)^{2}+\left( rsdCa \right)^{2}}$$

where “rsdMg” and “rsdCa” are the relative standard deviations of the Mg and Ca “counts per second” measured 5 times by the ICP-MS.

B) The External Reproducibility derives from multiple measurements of a known and gravimetrically prepared Standard Solution of Mg and Ca (SS) measured several times during the ICP-MS session. The relative standard deviation (rSD_SS_) of these measurements gives us the external reproducibility, which averages 13%.

The total relative error in Mg/Ca is estimated considering these two error sources and propagating them according to the law of uncertainty propagation for uncorrelated variables^1^:

$$Tot.Rel. Err. \left( \frac{Mg}{Ca} \right)= \sqrt{\left[ Mes.Rel. Err. \left( \frac{Mg}{Ca} \right) \right]^{2}+\left[ {rSD}_{SS} \right]^{2}}$$

The total absolute error in Mg/Ca ratios ($\Delta\left( \frac{Mg}{Ca} \right)$) is then calculated by multiplying the “Tot.Rel.Err. (Mg/Ca)” by each Mg/Ca ratio and divided by 100.

$$\Delta\left( \frac{Mg}{Ca} \right)=\frac{\left[ Tot.Rel. Err. \left( \frac{Mg}{Ca} \right) \right]*\frac{Mg}{Ca}}{100}$$

The absolute error associated to the SST ($\Delta T \left( ºC \right)$) has been estimated according to the law of uncertainty propagation for uncorrelated variables, that is, by partially deriving the equation for the involved terms^1^ that have an associated error; in this case, the Mg/Ca ratio and the error associated to the SST equation provided by Elderfield and Ganssen (2000)^2^. The resulting expression is:

$$\Delta T \left( ºC \right)=\sqrt{\left( \frac{10}{\frac{Mg}{Ca}}* \Delta\left( \frac{Mg}{Ca} \right) \right)^{2}+\left( \frac{10}{0.52}* 0,0085 \right)^{2}}$$

Therefore, the resulting average of $\Delta T \left( ºC \right)$ for the Core SW104-ND11 is ±1.45°C.

The absolute errors associated with a temperature change between two points ($\Delta\Delta T \left( ºC \right)$) have been calculated taking in to account each SST error ${(\Delta T}_{1}SST$ and ${\Delta T}_{2}SST$) involded in to the gradient (cooling or warming trend) and it has been applied the law of uncertainty propagation for uncorrelated variables^1^. The resulting expression is:

$$\Delta\Delta T \left( ºC \right)=\sqrt{{{(\Delta T}_{1}SST)}^{2}+{{(\Delta T}_{2}SST)}^{2}}$$

**S2. Criteria for the selection of the marine sites used for comparison**

The presented compilation of SST reconstructions has focussed in those records based on, the Mg/Ca ratio measured in planktonic foraminifera and the unsaturation alkenone ratio, covering the last 2kyr with a resolution and chronology good enough to resolve multi-centennial scale variability. This criterion has already cancelled the consideration of some available Mediterranean Holocene SST reconstructions that were produced with the objective to discuss longer timescales^3,4,5,6^. A previous compilation effort concentrated on the last 3kyr used alkenone-SST records from Mediterranean deltaic systems and showed a very complex regional pattern attributing it to local factors such as the nearshore inﬂuence of river runoff, human impact or wind system, producing a very distinctive signal to that from open sea sites^7^. Accordingly with these results, this study has excluded those records close to river runoff influence, such as Po and Ofanto Rivers in the Adriatic Sea, Rhone river in the Gulf of Lion, or under the direct influence of human impact and local oceanographic circulation^7,8,9^.

Consequently, the SST records chosen in this study aim to reflect wide regional conditions rather than the dominant influence of local factors that could mask the overall regional climate conditions. This selection consist on four records covering a W-E transect: an alkenone-SST record from the Alboran Sea^10^; a *G. bulloides* Mg/Ca-SST stack from north Minorca^11^; the new *G. ruber* Mg/Ca-SST record generated in this study and a composite of two alkenone-SST records from the Aegean Sea^12,13^. Any comparison between different geochemical proxies needs to have in consideration that these proxies do not reflect the same oceanographic conditions in terms of seasonality and/or water depth. In this regard, SST reconstructions based on Mg/Ca ratio in planktonic foraminifera has been interpreted to represent a narrower season^11,14^ than those reconstructed by alkenone measurements, which are interpreted as annual average temperatures^3,4,5,15^. Further individual criteria were applied for each of the chosen locations:

1. Both Mg/Ca and alkenone SST stack records were available for the north Menorca location^11^. This study has selected the *G. bulloides* Mg/Ca SST stack since, it is the one which presents larger SST oscillations while the alkenone-SST record, although presents comparable main trends, short term oscillations are within the error of the proxy. As discussed in the original manuscript^11^, the smoothed character of the Alk-SST record is interpreted to reflect the averaged intra-annual SST variability while Mg/Ca-SST record reflect better the variability within the main upwelling season, late winter early spring^11^.
2. For the Alboran Sea several alkenone and Mg/Ca records are available^5,6^ but the chosen record from site 434G^10^ is the one with higher resolution and more solid chronology for the considered period. A new high resolution record from a nearby location, ALB-2, is now available which also has an appropriate chronology^14^. This new *G. bulloides* Mg/Ca-SST record represents mostly the spring season which, at this location, is the season with strongest influence of the inflowing jet of north Atlantic waters^16^. Consequently, the alkenone-SST from site 434G was considered to better represent the averaged Mediterranean climate conditions that dominate along the whole year in the region.
3. For the eastern Mediterranean basin Mg/Ca-SST reconstructions are not available and we have focussed on those based on alkenones. In this case, taking is consideration the resolution and chronological controls of the available records, we have chosen the data published by Gogou et al. (2016)^12^ and Kontakiotis (2016)^13^ for the north Aegean Sea. This choice is also linked to the fact that this area is located at the same longitude position (core M2^12^: 40°05.15’N, 24°32.68’E; core SL_152^13^: 40°05.19’N, 24°36.65’E) than the *G. bulloides* Mg/Ca-SST stack from the Minorca basin^11^ (40°29’N, 3°37’E). An additional alkenone-SST reconstruction is available for the very eastern part of the Mediterranean basin (marine area of Israel) with a reasonable resolution and chronology^17^. But its inclusion in our compilation was rejected since the location is close to the Nile delta outflow, but it still remarkable that this record (Fig. S4), at lower resolution, validates the main trends observed in the Aegean SST-alkenone reconstructions including a prominent warming phase over the Roman Period (Fig. S4). This comparison supports the regional value of the selected records for the Eastern Mediterranean basin.


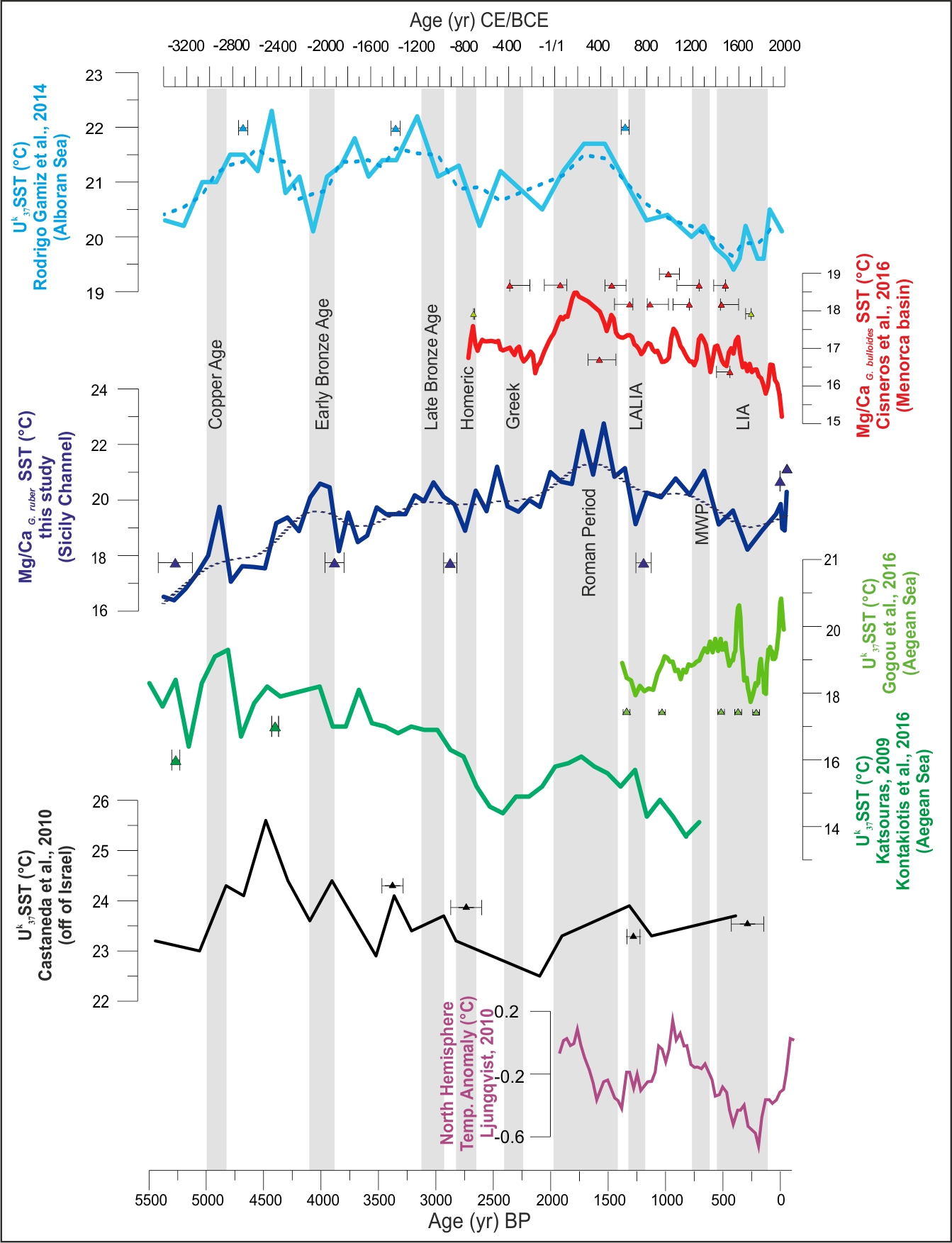


**Fig. S4 -** Comparison in time domain of the Mg/Ca *G. ruber* SST reconstruction of core SW104-ND11 (Sicily Channel, this work; the blue dashed line represents the 95% CI smoothed curve - Monte Carlo simulation), the SST-alkenone of Alboran Sea^10^ (with superimposed 3 points moving average), the Mg/Ca *G. bulloides* SST of Menorca Basin^11^, the SST-alkenone of Aegean Sea^12,13^ (SST data of the Aegean Sea^12^ is 3 points running average), the SST-alkenone of eastern Mediterranean^17^ with the north Hemisphere temperature reconstruction^18^. The grey bands show the main climate events documented in the Mediterranean basin and discussed in the text. Close to each record are the dating points with the error bars.

**References**

1. Taylor, B.N. and Kuyatt, C.E. Guidelines for Evaluating and Expressing the Uncertainty of NIST Measurement Results. *NIST Technical Note* **1297**, (1994).

2. Elderfield, H. & Ganssen, G. Past temperature and ^18^O of surface ocean waters inferred from foraminiferal Mg/Ca ratios. *Nature* **405**, 442–445 (2000).

3. Cacho, I. et al. Variability of the western Mediterranean Sea surface temperature during the last 25 000 years ans its connection with the Northern Hemisphere climatic changes. *Paleoceanography* **16**, 40–52, (2001).

4. Martrat, B. et al. Abrupt Temperature Changes in the Western Mediterranean over the Past 250, 000 Years. *Science* **80**, 306 (2004).

5. Martrat, B., Jimenezamat, P., Zahn, R., Grimalt, J. O. Similarities and dissimilarities between the last two deglaciations and interglaciations in the North Atlantic region. *Quaternary Science Review* **99**, 122–134, (2014).

6. Jiménez-Amat, P. & Zahn, R. Offset timing of climate oscillations during the last two glacial-interglacial transitions connected with large-scale freshwater perturbation. *Paleoceanography* **30**, 768– 788 (2015).

7. Jalali, B. et al. Deltaic and coastal sediments as recorders of Mediterranean regional climate and human impact over the past three millennia. *Paleoceanography and Paleoclimatology* **33**, 579–593 (2018).

8. Sicre, M.A. et al. Sea surface temperature variability in the North Western Mediterranean Sea (Gulf of Lion) during the Common Era. *Earth and Planetary Science Letters* **456**, 124-133 (2016).

9. Cascella, A. et al. Paleoclimate history of the last 2700 years in the Southern Adriatic Sea: Coccolithophore evidences. *The Holocene*, 1-12 (2019).

10. Rodrigo-Gámiz, M., Martínez-Ruiz, F., Rampen, S.W., Schouten, S., Sinninghe Damsté, J.S. Sea surface Temperature variations in the western Mediterranean Sea over the last 20 kyr: A dual-organic proxy (UK′37 and LDI) approach. *Paleoceanography* **29**, 87–98 (2014).

11. Cisneros, M. et al. Sea surface temperature variability in the central-western Mediterranean Sea during the last 2700 years: a multi-proxy and multi-record approach. *Climate of the Past* **12**, 849–869 (2016).

12. Gogou, A. et al. Climate variability and socio-environmental changes in the northern Aegean (NE Mediterranean) during the last 1500 years. *Quaternary Science Reviews* **136**, 209-228 (2016).

13. Kontakiotis, G. Late Quaternary paleoenvironmental reconstruction and paleoclimatic implications of the Aegean Sea (eastern Mediterranean) based on paleoceanographic indexes and stable isotopes. *Quaternary International* **401**, 28-42 (2016).

14. Català, A., Cacho, I., Frigola, J., Pena, L.D. & Lirer, F. Holocene hydrography evolution in the Alboran Sea: a multi-record and multi-proxy comparison. *Climate of the Past* **15**, 927–942 (2019).

15. Versteegh, G.J.M., de Leeuw, J.W., Taricco, C., Romer, A. Temperature and productivity influences on U37 K0 and their possible relation to solar forcing of the Mediterranean winter. *Geochemistry, Geophysics, Geosystems* **8**, Q09005 (2007).

16. Macias, D., Garcia-Gorriz, E., & Stips, A. The seasonal cycle of the Atlantic Jet dynamics in the Alboran Sea: direct atmospheric forcing versus Mediterranean thermohaline circulation. *Ocean Dynamics* **66**, 137–151 (2016).

17. Castañeda, I.S. et al. Millennial-scale sea surface temperature changes in the eastern Mediterranean (Nile River Delta region) over the last 27,000 years. *Paleoceanography* **25**, (2010).

18. Ljungqvist, F.C. A new reconstruction of temperature variability in the extra-tropical Northern Hemisphere during the last two millennia. Geografiska Annaler Series A. *Physical Geography* **92**, 339–351 (2010).
